# Neonatal estradiol influences regional gene expression for early sex-specific inflammatory responses in the 5xFAD mouse model

**DOI:** 10.1186/s13293-026-00948-z

**Published:** 2026-07-03

**Authors:** Phaedra R. Keller-Norrell, Brad T. Casali, Logan N. Yost, Erin G. Reed

**Affiliations:** 1 School of Biomedical Sciences, Kent State University, Kent, OH 44240 USA; 2 Department of Pharmaceutical Sciences, College of Pharmacy, Northeast Ohio Medical University, 4209 State Route 44, Rootstown, OH 44272 USA; 3 Student Research Experience, College of Arts and Sciences, Kent State University, Kent, OH 44240 USA

**Keywords:** Masculinization, Estradiol, 5xFAD, Microglia, X chromosome inactivation, Organizational effects, Sex-specific

## Abstract

**Background:**

Alzheimer’s disease (AD), the most prevalent form of dementia, exhibits a strong sex bias, with women comprising two-thirds of all patients, for reasons that remain unclear. Microglia, as the brain’s resident immune cell, are key players in AD pathogenesis and are increasingly understood as being sex-specific. However, the mechanisms underlying these differences, and how they may in turn contribute to distinct pathogenesis has not been well examined. Therefore, this study aimed to investigate the organizational role of neonatal estradiol (E2) in early-life sex-patterning of microglia for disease onset and progression.

**Methods:**

We assessed the effect of neonatal estradiol exposure on microglial and neuronal density, microgliosis, and gene expression related to microglial identity and responses using immunohistochemistry, qPCR, and ProteinSimple Jess on-capillary immunoblotting.

**Results:**

We find neonatal estradiol administration influences the expression of genes and proteins involved in inflammation and X chromosome inactivation, without affecting cellular composition of the cortex and hippocampus. Additionally, we observed genotype-dependent changes to the female reproductive cycle in E2 treated 5xFAD females.

**Conclusions:**

Overall, the results of the present study provide novel insight into the role of steroid sex hormones in neonatal microglial programing, and how this programming may set the stage for further sex- and disease-linked alterations later in life.

**Supplementary Information:**

The online version contains supplementary material available at https://doi.org/10.1186/s13293-026-00948-z.

## Abstract (plain language summary)

Women are disproportionally affected by Alzheimer’s disease (AD), the most prevalent form of dementia, for reasons that have yet to be defined. Classical AD pathology is accompanied by a robust immune response mediated by microglia, which exhibit sex-specific characteristics. As steroid sex hormones like estradiol (E2) drive lasting changes to the brain during neurodevelopment, and previous studies have identified that estradiol administration during early postnatal development can masculinize both brain and behavior, we investigated the impact of E2-mediated masculinization on microglia in AD-relevant brain areas. We report E2 does not alter brain cytoarchitecture in the hippocampus or cortex, instead driving changes at the molecular level, with subtle shifts in microglial gene expression and changes in the expression of the molecular machinery responsible for X chromosome inactivation (XCI). Taken together, these results suggest that neonatal steroid sex hormones may exert effects beyond classical sexually dimorphic brain regions through the regulation and maintenance of XCI for sex-specific immune responses, leading to biased disease onset and progression.

**Supplementary Information:**

The online version contains supplementary material available at https://doi.org/10.1186/s13293-026-00948-z.

## Highlights

The absence of changes to cytoarchitecture in the cortex and hippocampus suggests a potential limitation of cytoarchitectural change to the canonical sexually dimorphic brain nuclei.

Microglial gene expression is altered in response to neonatal estradiol treatment, when paired with the absence of cytoarchitectural changes, this suggests estradiol-mediated brain masculinization occurs primarily at the molecular level beyond the sexually dimorphic nuclei.

Neonatal estradiol changes patterns of X chromosome inactivation.

Changes to molecular messengers in X chromosome inactivation constitute a potential novel mechanism of action for the estradiol masculinization paradigm.

**Supplementary Information:**

The online version contains supplementary material available at https://doi.org/10.1186/s13293-026-00948-z.

## Background

Alzheimer’s disease is characterized by the deposition of extracellular amyloid, the accumulation of intracellular hyperphosphorylated tau proteins, and extensive neuronal atrophy. This is accompanied by robust microglial activation occurring in response to and perpetuating these disease processes [1–3]. While age is the single greatest risk factor for developing AD, females have a significantly increased lifetime risk of developing the disease, with a one-in-five chance, compared to the one-in-ten chance observed in men [4]. Though the mechanisms behind this bias remain poorly understood, a growing body of evidence suggests the bias in the induction and progression of AD is likely to be multifactorial, with environment, genetics, lifestyle, and individual biochemistry working in concert to shape AD risk across the lifespan [5].

One such biochemical difference that has been a central focus and highly debated for many years is the role of sex hormones, with multiple hallmark clinical trials highlighting the role of hormone replacement therapy (HRT) in AD [6–8]. HRT gained prominence within aging research as the loss of circulating sex hormones in menopause may also reduce neuroprotection and therefore be a potential risk factor for developing AD [9, 10]. While menopause does confer increased risk to AD patients, HRT is not an ideal therapy for everyone and is complicated by a potential temporal effect, where starting HRT fewer than ten years post-menopause is beneficial and has minimal risks, while therapy beginning more than ten years post-menopause increases risk of cognitive decline [6, 8]. These findings suggest a complex and nuanced role of hormones in both health and disease and warrants further investigation regarding the impact of hormones on biochemical processes across the lifespan.

In addition to their canonical role as immune cells responding to injury or potential pathogens, microglia are active in brain development and homeostasis [11–13]. During neonatal development, sequential surges of sex steroid hormones during a critical window organize and pattern the brain in a sex-specific manner [14]. Many of these early brain sex differences are driven by microglia, which are sensitive to early hormone pulses [14, 15]. In the highly sexually dimorphic medial preoptic area (mPOA), estradiol (E2) in the male brain increases microglial prostaglandin E2 (PGE2) for subsequent intensification of neuronal AMPAergic signaling and Spinophilin, ultimately amplifying dendritic spine density for male patterns of connectivity and observable behavioral changes, with E2-treated females exhibiting male sexual behavior and urination patterns [15].These hormonal effects during the critical period extend beyond behavior to include neonatal microglial phagocytic phenotype, with more phagocytic microglia in female hippocampi compared to age-matched male counterparts [13]. Moreover, they have life-long effects, as microglia retain their identity and sex-specific responses following depletion of host microglia and transplant into a recipient of the opposite sex [16].Impeding microglial PGE2 signaling feminizes males, indicating the essential role of microglia as mediators in brain sexual differentiation [15, 17].

## Methods

### Animal husbandry

5xFAD mice were obtained from Jackson Laboratories (034840-JAX) [18] and a colony was maintained in-house at the Northeast Ohio Medical University (NEOMED) Comparative Medicine Unit in accordance with IACUC protocol (USDA #31-R-0092, AWA #A3474-01). Animals were ear notched for identification, and tissue was sent to Transnetyx (Cordova, TN, USA) for genotyping using a proprietary probe for humanized PSEN1 and an internal control. Animals were housed in microisolator barrier housing (no more than 5 animals per cage) and given ad libitum access to food and water. Following E2 treatment, cage bedding was changed by the investigator seven days post-treatment cessation and disposed of as regulated biohazardous waste.

### Tissue collection

2-month-old 5xFAD and WT littermate control animals were euthanized via an overdose of Isoflurane gas anesthesia followed by cervical dislocation. Tissues were collected for biochemical analysis: trunk blood was collected and stabilized with EDTA, and brains were bisected with the right hemisphere proceeding to microdissection where the hippocampus and cortex were isolated and snap frozen on dry ice. The left hemisphere was drop-fixed overnight in 4% paraformaldehyde in PBS, before being transferred to 30% sucrose prior to cryosectioning. Tail snips were collected for genotype verification by Transnetyx.

### Estradiol administration

Beta-Estradiol 3-benzoate (Sigma Aldrich, #E8515-200MG) was compounded to a final concentration of 50µg/mL, using 1mL absolute ethanol to solubilize and suspended in 49 mL corn oil (Sigma Aldrich, C8267-500ML) to generate stock aliquots. Stock aliquots were further diluted (250 µL stock in 750 µL corn oil) to achieve the desired 50 µg/mL dose. Three doses of 2.5 µg (50µL stock/injection) estradiol were administered subcutaneously between the shoulders on postnatal days 2, 5, and 8, as previously described [15].

### Immunohistochemistry

Brains were transferred from sucrose, embedded in Optimum Cutting Temperature compound (OCT, Ted Pella), and stored in cassettes at -80°C until sectioning. Serial 30µm sagittal sections were collected using a Leica CM 1950 cryostat, and placed into storage cryoprotectant (30% ethylene glycol, 30% glycerol in PBS) until staining. Four sections per animal were visually selected to form a representative sample across the cortex, moving from medial to lateral. Tissue was stained as we previously reported using rabbit monoclonal Iba1 (FujiFilm Wako Pure Chemical Corporation #18–28,523, 1:1000) and mouse monoclonal NeuN (Abcam #ab104224, 1:1000) antibodies [19, 20]. An additional four sections per animal were stained with goat monoclonal Iba1 (Novus Biologicals, #NB100-1028) and rabbit monoclonal CD68 (Cell Signaling Technologies, #97778S) antibodies. Stained sections were mounted to SuperFrost Plus microscope slides (Fisher Scientific #12–550-15), coverslipped using Prolong Gold Antifade mounting medium with DAPI (ThermoFisher Scientific #P36931), and allowed to dry overnight prior to imaging.

### Microscopy

**Fig. 1:**
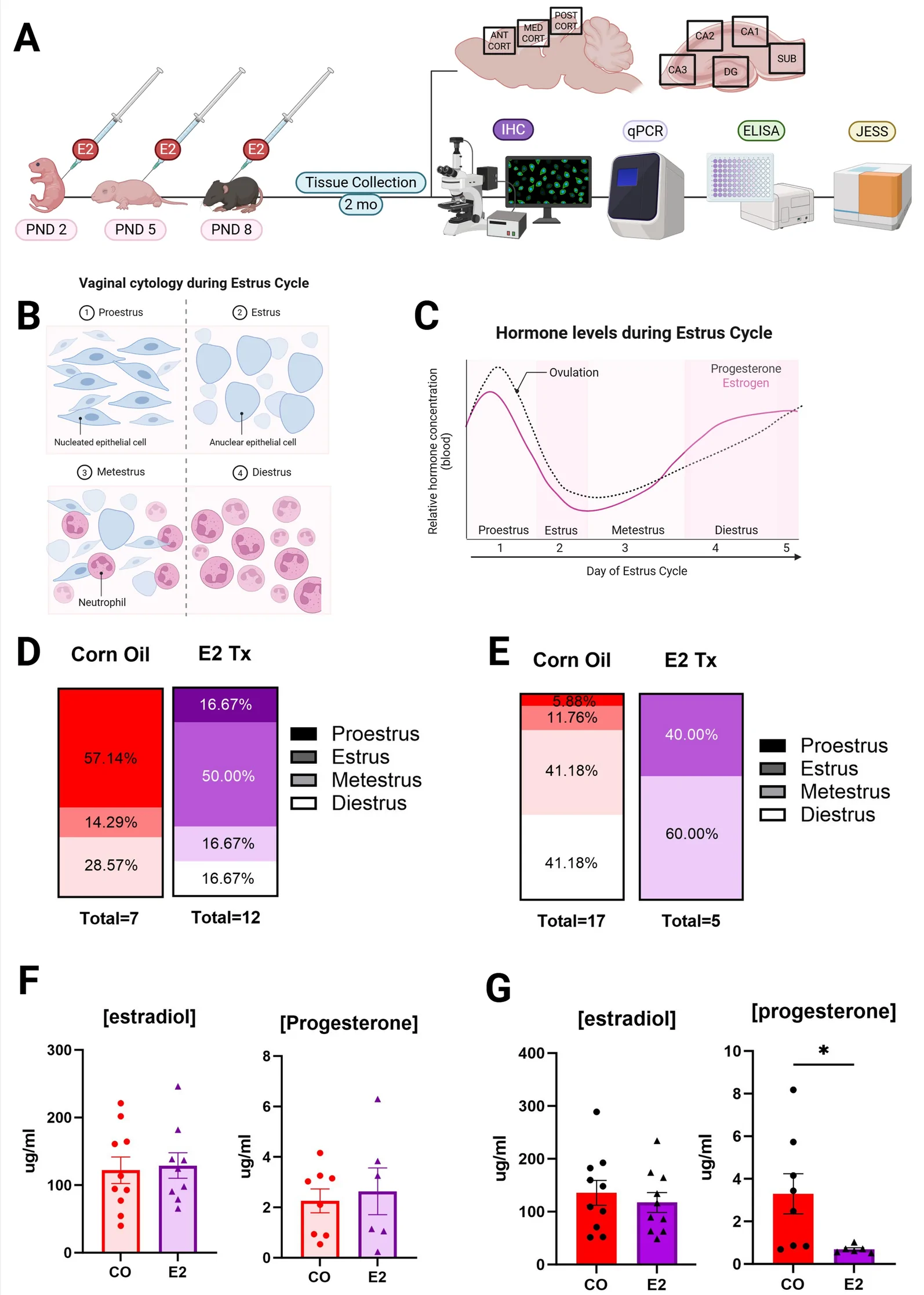
Systemic effects of the E2 masculinization paradigm in wild type and 5xFAD animals. (**A**) Study timeline and IHC imaging locations. (**B**) Schematic of vaginal cytology over the estrus cycle used to stage vaginal lavages at time of harvest. (**C**) Depiction of hormone fluctuations during the estrus cycle. The distribution of wild-type (WT; **D**) and 5xFAD (**E**) corn oil (CO)- and E2- treated female mice across the estrus cycle based on staging via vaginal lavage at euthanasia. Plasma concentrations of estradiol and progesterone in WT (**F**) and 5xFAD (**G**) CO- and E2-treated female mice, determined by ELISA. Graphs represent mean ± SEM, where n = 6–10 mice/group. Created in BioRender. Norrell, P. (2026) https://BioRender.com/cav8ueu

Sections were imaged using a Zeiss AxioZoom V16 at 125 × magnification or 32–40 × for lateral hippocampal sections, and a Leica DM4B at 40 × magnification. Images captured for Sholl analysis were imaged at 100 × on the Leica DM4B. For hippocampal imaging, z-stacks were acquired spanning the thickness of the section, visually confirmed at time of imaging by loss of focus at the top and bottom of the stack. Five images were taken in the hippocampus, representing all hippocampal subregions (CA1, CA2, CA3, Dentate Gyrus (DG), and the subiculum). Sholl imaging focused on CA3 of the hippocampus, as sex-linked differences in microglial morphology have been previously reported in this region [21]. See Fig. 1A for graphical depiction of ROIs. Visual landmarks were used in conjunction with the Allen Institute Mouse Brain Reference Atlas (available from https://atlas.brain-map.org/) to confirm image spacing in the cortex and regional specificity in the hippocampus [22].In the cortex, three images (anterior, medial, posterior) were acquired without Z-stack, using visual confirmation of focus within the majority of the field of view to establish a focal plane prior to image capture. Images focused on cortical layer V, as this layer is particularly vulnerable to neuronal atrophy and cell death in the 5xFAD [23].

### Image analysis

Max projections from all captured z-stack images, except for Sholl, were analyzed using FIJI ImageJ. For cortical NeuN counts, a threshold was manually established, and cells were separated using the “watershed” function prior to counting, with a 0.2µm exclusion on the edges of the image. For hippocampal NeuN, the thickness of the pyramidal cell layer was determined by averaging the length of fifteen independent line segments spaced across the sample and reported as thickness in microns. For Iba1 counts (both cortical and hippocampal), images were manually thresholded, and an overlap mask of Iba1 positive cells and DAPI positive cells were created, such that only double-positive cells were counted. Area of the double-positive mask was then divided by the total sample area to determine percent immunoreactive area. For CD68 percent area, an overlap mask of CD68 and Iba1 was created, and percent immunoreactive area was calculated as described above. Sholl analysis was completed using methods previously described [21, 24]. All image analysis results are relative to the area (in microns) of the acquired image(s).

#### qPCR

Brain microdissections were stored at -80°C until homogenization. Tissue was homogenized using a rotor–stator homogenizer in ice-cold homogenization buffer (1M Tris, 8.5% sucrose [w/v], 0.5 M EDTA, and 0.2 M EGTA in ultrapure water, pH 7.4) supplemented with fresh protease inhibitor cocktail (Sigma #P3480; 1:100 dilution). A fraction of the homogenate was suspended in RNA STAT-60 (Amsbio #CS-110) and snap frozen until time of processing. RNA was purified from the RNA STAT-60 containing samples using the Invitrogen PureLink RNA Mini Kit (#12,183,025) and converted to cDNA using the Applied Biosystems High-Capacity RNA to cDNA Kit (#4,387,406). The extracted cDNA was pre-amplified prior to use in qPCR analysis (ThermoFisher Applied Biosystems TaqMan PreAmp Master Mix #4,391,128). The following TaqMan probes (ThermoFisher) were used for both pre-amplification and analysis (*IL-1α*, Mm00439620_m1; *Akt1s1*, Mm01203150_g1; *CD36*, Mm00432403_m1; *Ccl22*, Mm00436439_m1; *IL-1β*, Mm00434228_m1; *CXCL1*, Mm04207460_m1; *Trem2*, Mm04209422_m1; *CXCL10*, Mm00445235_m1; *GFAP*, Mm01253033_m1; *Ifnγ*, Mm01168134_m1; *CXCL12*, Mm00445553_m1; *Aif1* (*Iba1*), Mm00479862_g1; *CXCL2*, Mm00436450_m1; *Xist*, Mm01232884_m1;*Mid1*, Mm04933166_m1; *Iqsec2*, Mm02344191_m1;*Eif2s3x*, Mm01236979_g1; *Ubqln2*, Mm00834570_s1; *Huwe1*, Mm00615533_m1; *Cx3Cr1*, Mm02620111_s1; *Kdm6a*, Mm00801998_m1; *P2ry12*, Mm01950543_s1; *Itgax*, Mm00498701_m1; *Csf1r*, Mm01266652_m1; *Clec7a*, Mm01183349_m1; *Cst7*, Mm00438351_m1; *Kdm5c*, Mm00840032_m1). The average Ct of two endogenous housekeeping genes (*18s*, Hs99999901_s1; and *GAPDH*, Mm99999915_g1) was used to determine dCt, and data is presented as raw dCT values, normalized across rows. All qPCR experiments were run in triplicate, with n ≥ 8 mice/group, though n sizes depicted in figures may vary due to removal of outliers, or lack of target detection.

#### Jess

An aliquot of whole cortical or whole hippocampal homogenate was centrifuged (5000 RCF for 10 min at 4°C) to pellet cellular debris. The resulting supernatant was aliquoted into clean 0.2 mL falcon tubes, snap frozen on dry ice, and stored at -80°C until time of use. Protein concentration was determined via BCA and was used to normalize the final Jess sample concentration to 0.2mg protein/capillary. The Jess platform is an on-capillary immunoblotting system that provides size-based measurement of target proteins like a Western Blot. Chemiluminescent spectra are compared against the molecular weight of a target protein, which is determined using manufacturer-supplied biotinylated standards of known molecular weight (BioTechne, PS-ST03EZ-8) [25, 26]. This chemiluminescent peak area is then normalized to total sample protein using the ProteinSimple Total Protein (DM-TP01) and RePlex (RP-001) modules. Samples were prepared in accordance with ProteinSimple protocol, utilizing the ProteinSimple anti-rabbit detection module (DM-001), and 66–440 kDa separation module (SM-W005). Antibodies to proteins involved in X-chromosome inactivation – Smchd1 (200 kDa, Novus Biologicals, NBP1-82,978) and Dnmt3b (105 kDa, Cell Signaling Technology, 48488S) – were used at 1:20 and 1:10 respectively. Results are reported as the total chemiluminescent peak area, normalized to total protein.

#### ELISA

Trunk blood was centrifuged at 15,000 RCF for ten minutes at 4°C to pellet red blood cells. The plasma supernatant was transferred to a new, clean 0.5mL Eppendorf tube, flash frozen on dry ice, and stored at -80°C. Plasma levels of E2 and progesterone (reported in µg/mL) were assayed using an Estradiol Parameter Assay Kit (R&D Systems, BioTechne #KGE014) and Progesterone Assay Kit (BioVendor #RTC008R). Plates were read using a SpectraMax M5 Multi-Mode plate reader (Molecular Devices #0310–5625) and analyzed according to kit specified parameters using SoftMax Pro, Version 6.5.1.

### Vaginal cytology

Vaginal lavages were taken at time of sacrifice using 50 µl of PBS and mounted to SuperFrost Plus microscope slides (Fisher Scientific #12–550-15). The mounted samples were allowed to air dry overnight before staining with Crystal Violet (SigmaAldritch #C0775-25G). Stained samples were allowed to air dry once more overnight before coverslipping with Permount mounting medium (Fisher Chemical # SP15-500). Lavages were visually inspected at 100 × using a brightfield microscope and staged based on cornification of vaginal epithelium and presence/absence of white blood cells [27].

### Statistical analysis

Data were analyzed by one-way analysis of variance (ANOVA) with Tukey’s multiple comparisons post hoc test, or unpaired t-test with Welch’s correction (used only for sex steroid ELISAs). Bar graphs represent mean ± standard error of the mean (SEM), while heatmaps represent raw qPCR ΔCT values, normalized across rows in BioRender Graph. Significance overlays depicted on heatmaps are taken from One-Way ANOVA of each respective gene. Jess heatmaps represent average chemiluminescent peak area normalized to total protein, normalized across rows in BioRender Graph. Significance indicators depicted on Jess heatmaps are taken from One-Way ANOVA of each respective protein, with WT and 5xFAD groups analyzed independently of each other to maintain statistical power. Statistical significance was set at p < 0.05. Outliers were removed from all datasets via Grubb’s test (α = 0.05). All analyses were performed using GraphPad Prism version 11.0.0 for Windows (GraphPad Software, Boston, MA, USA).

## Results

### E2 does not alter vaginal cytology or circulating hormone levels in E2 treated mice

To determine whether neonatal sex hormones contribute to divergent microglial responses and therefore sex-specific disease onset and trajectory, we treated 5xFAD mice and their wildtype littermates with E2. E2 administration to masculinize female rodents (Fig. 1A) is a widely used paradigm within the field of sex differences research [15, 28, 29]. While the effects on behavior have been well-described, the associated cellular and molecular changes are only beginning to be understood, and little is known regarding the systemic effects of E2 masculinization beyond the brain. We first aimed to assess potential systemic impacts of E2 masculinization, as microglia are intimately tied to the immune and neuroendocrine systems, and thus, may drive systemic changes to reproductive physiology when masculinized [30, 31]. We began by investigating potential alterations to vaginal cytology via vaginal lavage (Fig. 1B) and hormone levels (Fig. 1C) at time of sacrifice, as previous studies have observed prolonged cornification (persistence of anuclear epithelial cells) in response to treatment with estradiol or synthetic analogues [32, 33]. On average, the rodent estrus cycle lasts five days and consists of four phases: proestrus, estrus, metestrus, and diestrus [27]. Diestrus is the longest of the four phases, lasting up to 48 h, and is accompanied by increasing progesterone levels [34]. Upon exiting diestrus, females enter proestrus, where progesterone levels peak, while circulating estradiol levels decline [27, 34, 35]. Proestrus lasts up to 24 h, and circulating estradiol levels continue to decline prior to the estrus phase. Estrus occurs just after ovulation and is characterized by relatively stable levels of progesterone and estradiol before the transition to metestrus, where levels of progesterone begin to rise before the transition to diestrus, and the cycle repeats. At sacrifice, E2 administration had no significant impact on vaginal cytology of WT (Fig. 1D) or 5xFAD (Fig. 1E) female mice compared to their corn oil (CO)-treated counterparts. Though the proportion of animals in metestrus and diestrus was decreased in WT E2-treated females (Fig. 1D), this difference is likely attributable to an over-representation of these phases, as we did not track estrus prior to sacrifice. This trend was not replicated in 5xFAD E2-treated females (Fig. 1E) further supporting that any alterations in estrus phase distribution were attributable to lack of tracking and stratification prior to sacrifice. We also measured circulating levels of E2 and progesterone via ELISA to assess the impact of E2 treatment on endogenous hormone levels. There were no significant differences in either hormone level with E2 administration in WT females (Fig. 1F). Similarly, there was no difference in estrogen levels in 5xFAD females, but progesterone was significantly decreased in E2-treated 5xFAD females, likely attributable to the over-representation of low progesterone estrus phases (i.e. estrus and metestrus) in the sample at time of sacrifice. Taken together, these findings indicate a potential ovary-independent effect of E2 treatment, as reproductive physiology is spared.

### E2 does not significantly alter cortical or hippocampal cytoarchitecture

Previous studies have characterized sexually dimorphic nuclei in the brain, with particular attention paid to increases in neuronal density and volume of the mPOA, the medial amygdala, and the bed nuclei of the stria terminalis in males [36].However, brain sexual dimorphism is still hotly debated. While human functional imaging studies show no significant differences in volume of targeted brain regions between the sexes, rodent stereology studies indicate sexual dimorphism in multiple brain regions that persists across the lifespan [37, 38]. Therefore, it is imperative to continue investigating brain sexual dimorphism, especially within the current masculinization paradigm, to elucidate the impact of E2 beyond the canonical sexually dimorphic nuclei. To do so, we performed immunohistochemistry (IHC) for neurons and microglia using NeuN and Iba1, respectively, as both are targeted by E2 masculinization [14].

**Fig. 2:**
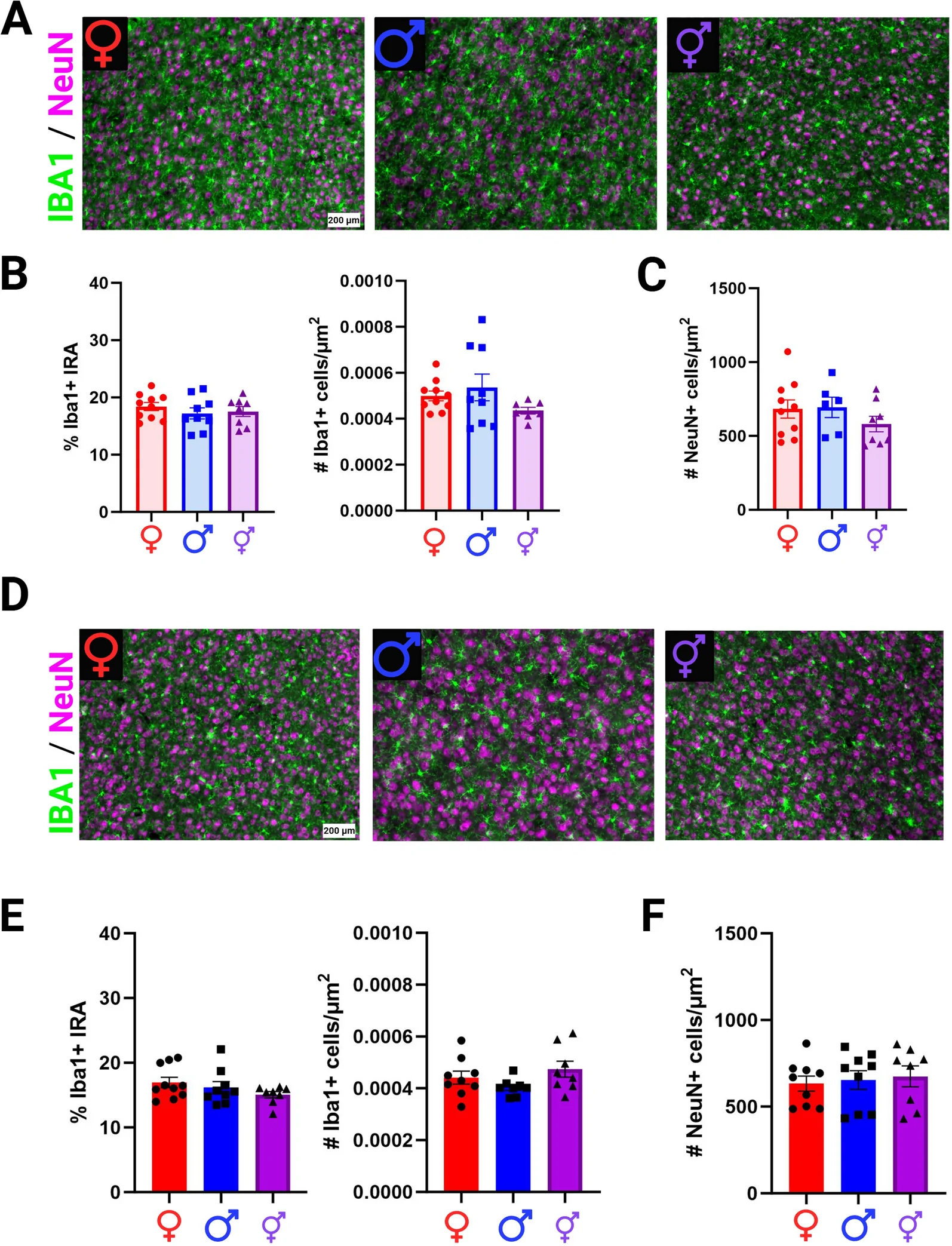
E2 administration does not alter cortical cytoarchitecture. (**A**) Representative images of NeuN (purple) and Iba1 (green) immunohistochemistry in the cortex of 2-month-old WT mice. Images depict the anterior cerebral cortex, with a focus on Layer V neurons. Quantification of the percent Iba1 + immunoreactive area and microglial cell density (**B**) and NeuN + cell density (**C**) across the cortex. (**D**) Representative NeuN (purple) and Iba1 (green) IHC in the cortex of 2-month-old 5xFAD mice. Images depict the anterior cerebral cortex, with a focus on Layer V neurons. Quantification of Iba + percent immunoreactive area and microglial cell density (**E**) and NeuN + cell density (**F**) in the 5xFAD cortex. Scale bars indicate 200 µm. Graphs represent mean ± SEM, where n = 8–10 mice/group. Created in BioRender. Norrell, P. (2026) https://BioRender.com/xwcpr4t

In cortical layer V, wild type (WT) CO- and E2-treated females exhibited no significant differences in Iba1 + immunoreactive area compared to CO-treated WT male littermates (Fig. 2A, B). As immunoreactive area is comprised of both microglial number and shape, we evaluated microglial density. CO-treated WT female and male mice exhibited comparable cortical microglial densities, with no effect of E2 treatment (Fig. 2B).

While total neuron number is known to be increased in the male mPOA, and mPOA morphology is known to be masculinized during the critical period, previous studies have not reported the impact of E2 masculinization on neuronal number/density beyond the canonical sexually dimorphic nuclei [14, 15, 39]. Therefore, we investigated NeuN + cell density in cortical layer V, and found no significant differences in NeuN + cell number between WT groups (Fig. 2C), indicating that E2 treatment does not have global effects within the CNS, suggesting that the morphological changes observed in previous studies are limited to the canonical sexually dimorphic brain nuclei [14, 15].

These findings were replicated in 5xFAD animals (Fig. 2D, E, F), which is unsurprising, as pathology in two-month-old 5xFAD animals is largely limited to the subiculum, and therefore, increases in percent immunoreactive area or total microglial cell number, and subsequent neuronal atrophy, have yet to occur. Analyses of subicular NeuN + cell density further revealed no significant differences between groups, indicating no significant impact of pathology on subicular NeuN + cell populations (Supplementary Fig. 1), further indicating that genotype-linked neuronal atrophy had yet to occur.

**Fig. 3:**
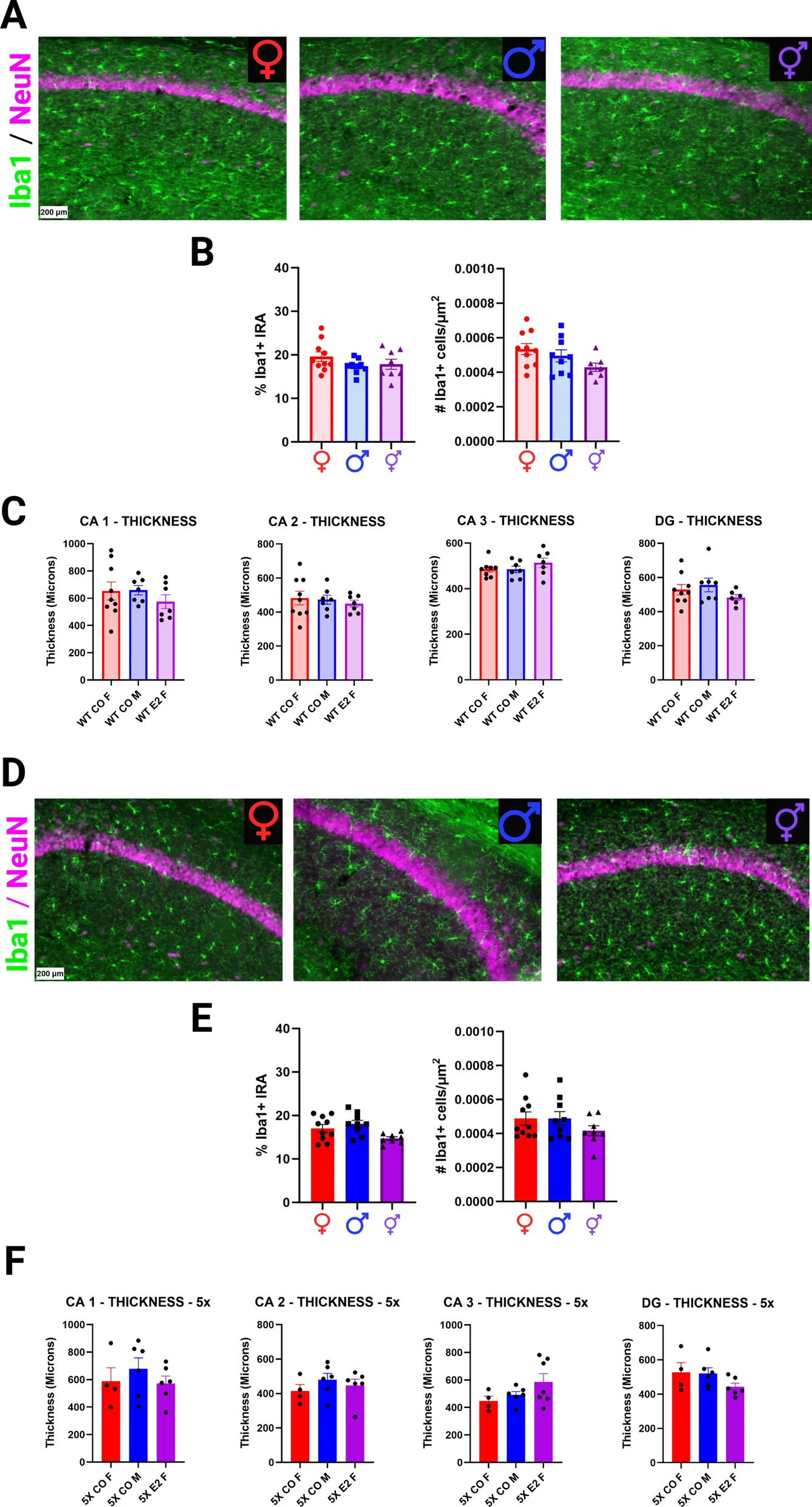
E2 administration does not alter hippocampal cytoarchitecture. (**A**) Representative images of NeuN and Iba1 IHC in the WT hippocampus depicting CA1. Quantification of the percent Iba1 + immunoreactive area microglial cell density (**B**) and NeuN + pyramidal layer thickness across hippocampal regions (**C**) across the WT hippocampus. (**D**) Representative images of NeuN and Iba1 immunohistochemistry in the 5xFAD hippocampus. Images depict CA1. Quantification of Iba + percent immunoreactive area and microglial cell density (**E**) and NeuN + pyramidal cell layer thickness (**F**) across the 5xFAD hippocampus. Scale bars indicate 200µm. Graphs represent mean ± SEM, where n = 4–10 mice/group. Created in BioRender. Norrell, P. (2026) https://BioRender.com/cd76vd2

We next assessed the hippocampus due to its relevance in AD, and because microglial density is increased in the hippocampus relative to the cortex, thus creating a potential for increased sensitivity to E2 masculinization [40]. Furthermore, microglia exhibit a high degree of regional heterogeneity, possibly driving region-specific responses to masculinization [40]. In WT animals, Iba1 + reactivity was consistent with that of the cortex, with no significant differences observed between groups (Fig. 3A, B). Similarly, there were no significant differences in microglial density (Fig. 3B). We also assessed pyramidal layer thickness across the four major hippocampal subregions, CA1, CA2, CA3, and the dentate gyrus (DG), to investigate any potential morphological changes to neuronal cell populations. We found no significant difference in pyramidal layer thickness between groups (Fig. 3C).

These findings were again replicated in 5xFAD animals (Fig. 3D, E and F), further suggesting that morphological and cytoarchitectural changes driven by of E2 treatment are localized to the sexually dimorphic brain nuclei, and that changes beyond these nuclei may be occurring at the ultrastructural or molecular level. Therefore, we next sought to determine if microglial morphology and activation states were altered.

### *E2 treatment does not alter microglial morphology in WT or 5xmice*

**Fig. 4:**
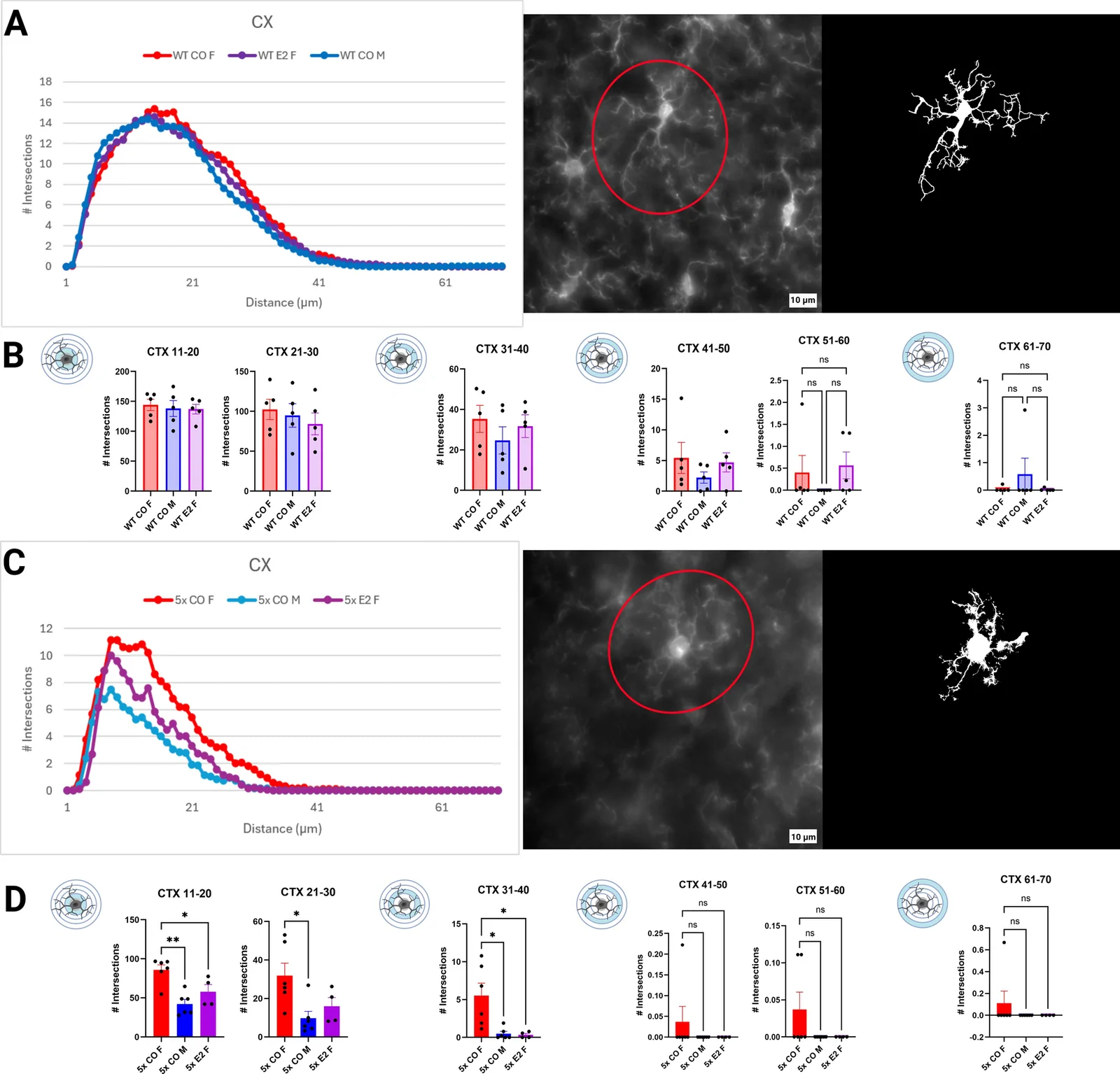
E2 administration alters cortical microglial morphology in a 5xFAD-dependent manner. (**A**) Sholl analysis of microglia from the WT cortex, focusing on cortical layer V. The average number of intersections (from 1-70µm from center of soma) is plotted for each group. Representative images (original image left with microglia selected for analysis circled in red, processed image right, scale bar indicating 10µm) depict microglia from the WT CO F anterior cortex. (**B**) Average number of intersections across the highest density sampled radii. (**C**) Sholl analysis of microglia from the 5xFAD cortex, focusing on cortical layer V. The average number of intersections (from 1-70µm from center of soma) is plotted for each group. Representative images (original image left with microglia selected for analysis circled in red, processed image right, scale bar indicating 10µm) depict microglia from the 5xFAD CO F anterior cortex. (**D**) Average number of intersections across the highest density sampled radii. One-way ANOVA with Tukey’s multiple comparisons post hoc indicated. * p < 0.05, ** p < 0.01 compared to 5x CO female and/or 5x CO male. Created in BioRender. Norrell, P. (2026) https://BioRender.com/vkury94

We next sought to investigate potential alterations to microglial ultrastructure, as microglial morphology is highly sexually dimorphic, and while morphology is not a direct indicator of activation state, it can provide insight into general microglial response [41, 42]. In early life, male microglia are primed for inflammatory responses, and retain their amoeboid morphology slightly longer than microglia from age-matched female counterparts [43]. In adolescence and adulthood, this trend is reversed, as female microglia become primed for pro-inflammatory responses, and males trend toward slightly anti-inflammatory responses [44, 45]. To assess potential E2-driven changes to microglial morphology, we performed Sholl analysis as previously described [21, 24]. The number of branches were averaged every ten microns, with the highest density intervals (i.e. 11–20 µm, 21–30 µm, 31-40µm, 41-50µm, 51-60µm, and 61-70µm) taken for analysis. In the cortex, we found no significant differences in the number of intersections across all sampled radii (Fig. 4A, B). Additional morphometrics were also analyzed, with the critical radius (the radius in which the highest number of intersections occur) being significantly farther from the center of the soma in WT CO females compared to WT CO males. This is a sex difference that is well documented, and is potentially indicative of the slightly more amoeboid morphology characteristic of male microglia in development and adolescence (Supplementary Fig. 2) [46]. Interestingly, in the 5xFAD, we observed masculinization of the number of intersections in the 11-20µm and 31-40µm intervals, suggesting a shift toward the male morphology (Fig. 4C, D). These differences were observed in conjunction with significant masculinization of the sum of intersections, mean intersections, and number of intersecting radii, suggesting broad masculinization of microglial morphology and branch complexity (Supplementary Fig. 3).

**Fig. 5:**
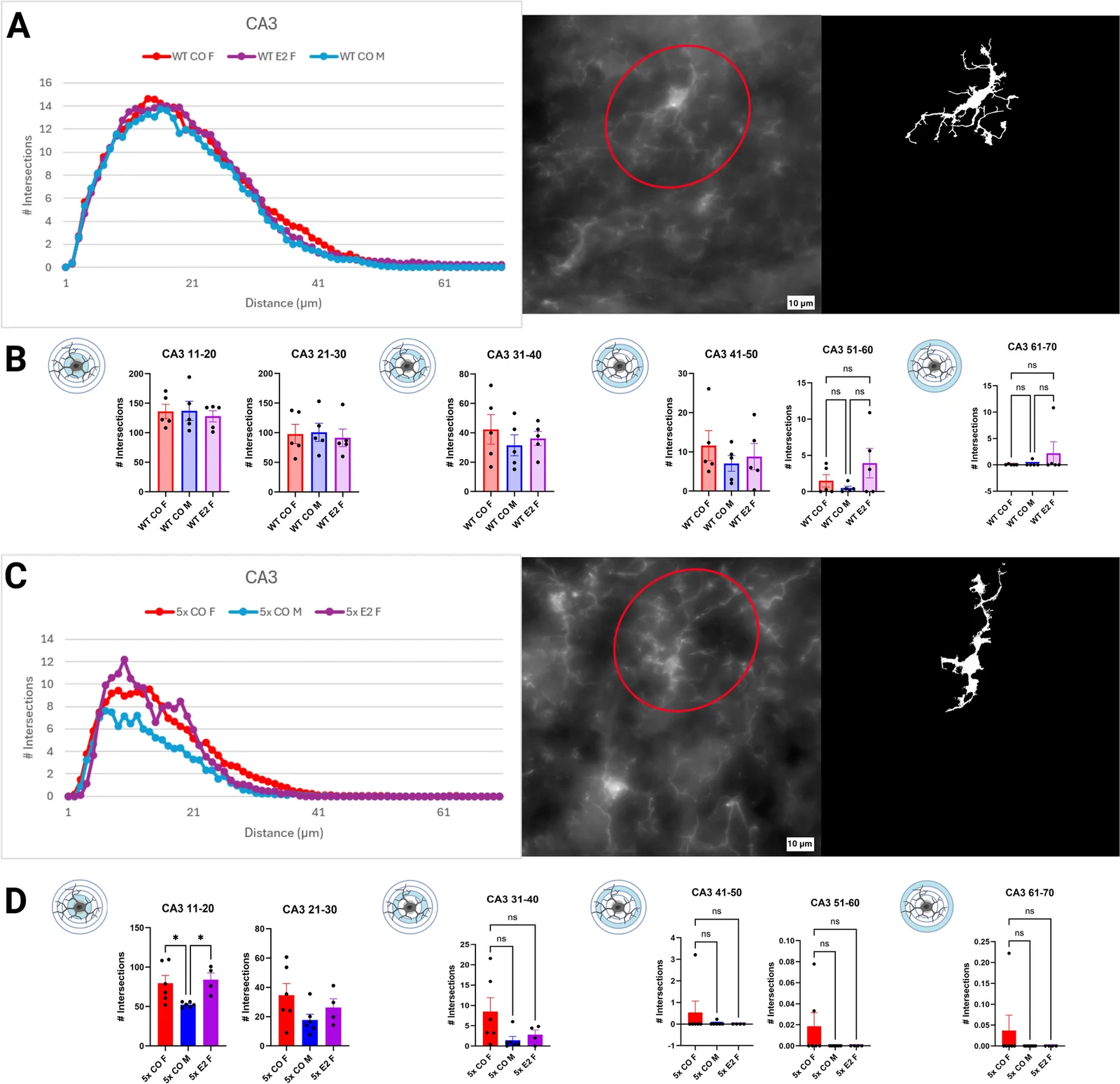
E2 administration does not masculinize microglial morphology in the WT or 5xFAD hippocampus. (**A**) Sholl analysis of microglia from CA3 of the WT hippocampus. The average number of intersections (from 1-70µm from center of soma) is plotted for each group. Representative images (original image left with microglia selected for analysis circled in red, processed image right, scale bar indicating 10µm) depict microglia from a WT CO treated female. (**B**) Bar graphs depict the average number of intersections within the highest branching density intervals. (**C**) Sholl analysis of microglia from CA3 of the 5xFAD hippocampus. The average number of intersections (from 1-70µm from center of soma) is plotted for each group. Representative images (original image left with microglia selected for analysis circled in red, processed image right, scale bar indicating 10µm) depict microglia from a 5xFAD CO treated female. (**D**) Bar graphs depict the average number of intersections within the highest branching density intervals. Created in BioRender. Norrell, P. (2026) https://BioRender.com/oamqxwm

Within the hippocampus, we focused on the CA3 as microglia in this region exhibit a greater degree of sexual dimorphism than those found in other non-canonically sexually dimorphic brain nuclei [21]. We found no significant differences in the number of intersections between groups in WT animals (Fig. 5A, B) suggesting E2 administration does not alter microglial morphology in WT animals beyond the sexually dimorphic brain nuclei. In 5xFAD animals, significant differences were present in the number of intersections between 11-20µm (Fig. 5C, D) however, this difference was not attributable to the masculinization paradigm. We also observed significant differences in the maximum number of intersections per radius in 5xFAD E2 treated females relative to their CO treated male and female counterparts, though the mechanism underlying this difference remains unclear (Supplementary Fig. 5). 5x CO treated females also exhibited an increase in the number of intersecting radii, indicative of the increased ramification that is typical of the female microglial phenotype [46].

**Fig. 6:**
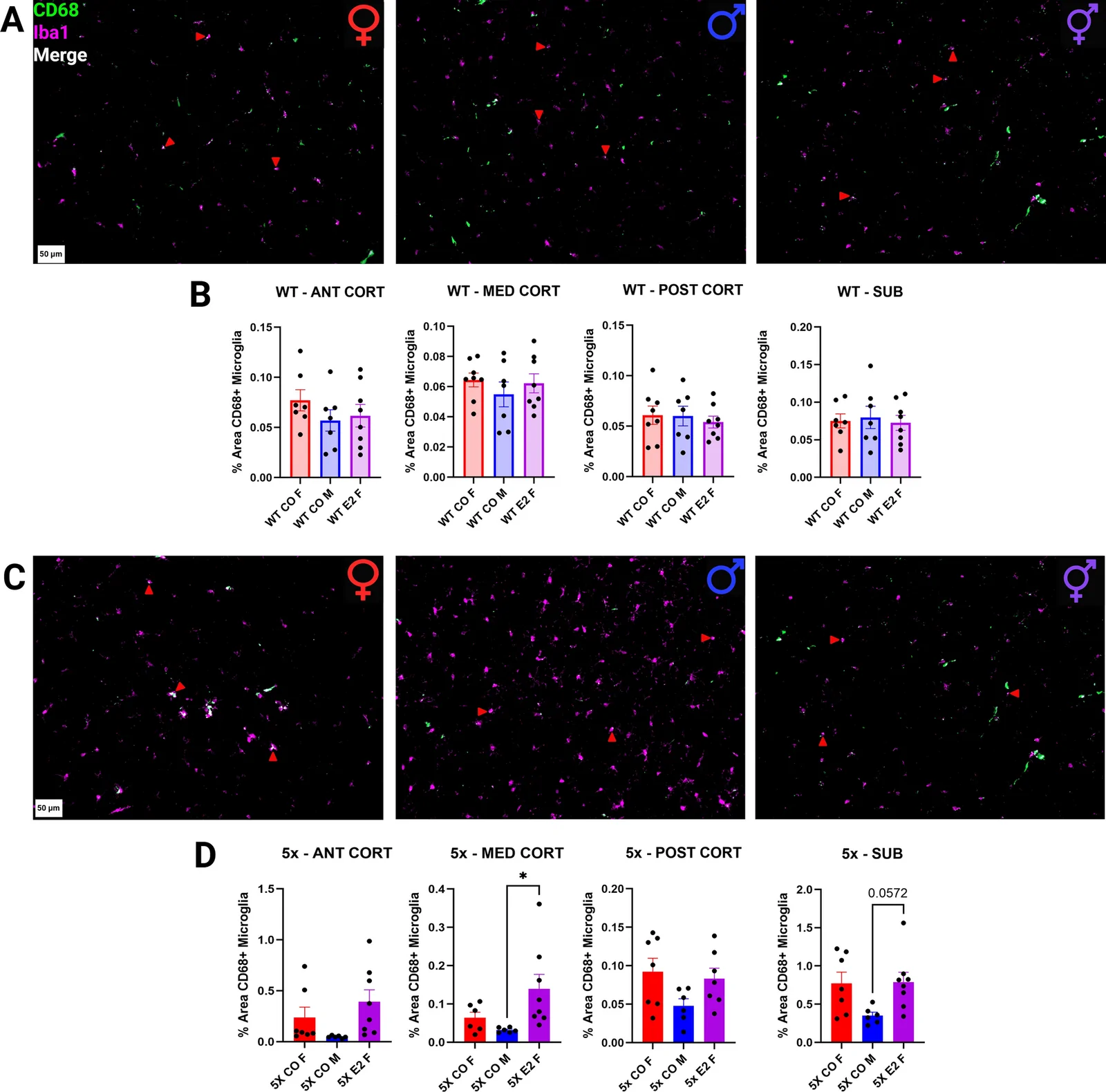
E2 administration does not impact microglial phagocytosis in the WT or 5xFAD cortex. (**A**) Representative images of CD68 (green) and Iba1 (purple) in WT anterior cortices, with a focus on cortical layer V. Scale bar indicates 50 µm. Red arrowheads indicate cells where colocalization of Iba1 and CD68 is present. (**B**) Percent area of CD68 + microglia across all regions of interest in WT animals. (**C**) Representative images of CD68 (green) and Iba1 (purple) in 5xFAD anterior cortices, with a focus on cortical layer V. Scale bar indicates 50 µm. Red arrowheads indicate cells where colocalization of Iba1 and CD68 is present. (**D**) Percent area of CD68 + microglia across all regions of interest in 5xFAD mice. Created in BioRender. Norrell, P. (2026) https://BioRender.com/v2amudv

**Fig. 7:**
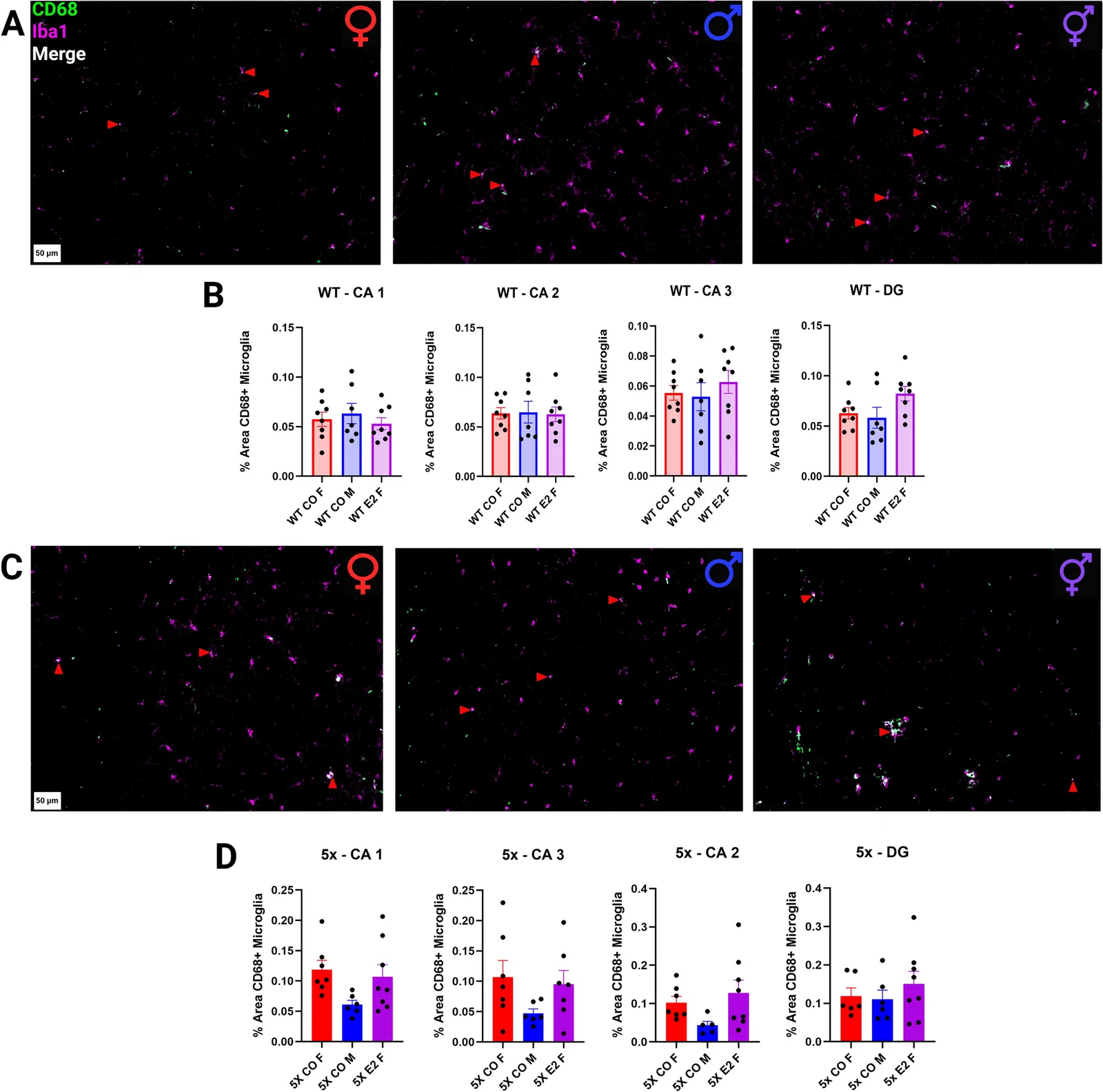
E2 administration does not impact microglial phagocytosis in the WT or 5xFAD hippocampus. (**A**) Representative images of CD68 (green) and Iba1 (purple) in CA1 of the WT hippocampus. Scale bar indicates 50 µm. Red arrowheads indicate cells where colocalization of Iba1 and CD68 is present. (**B**) Percent area of CD68 + microglia across all regions of interest in WT animals. (**C**) Representative images of CD68 (green) and Iba1 (purple) in CA1 of the 5xFAD hippocampus. Scale bar indicates 50 µm. Red arrowheads indicate cells where colocalization of Iba1 and CD68 is present. (**D**) Percent area of CD68 + microglia across all regions of interest in 5xFAD mice. Created in BioRender. Norrell, P. (2026) https://BioRender.com/yzi3ssy

Sex differences in microglial activation states and phagocytosis have also been reported, with age-dependent changes in phagocytosis indicated by CD68, a lysosomal marker commonly used to quantify microglial engulfment [21, 47, 48]. Therefore, we determined the impact of E2 treatment on microglial phagocytic phenotype by quantifying the percent area of CD68 + microglia in the cortex (Fig. 6) and hippocampus (Fig. 7). We observed no significant differences between groups in WT animals in either brain region (Figs. 6 and 7). In 5xFAD animals, there tended to be more CD68 + microglia in the cortex (Fig. 6C, D) and hippocampus (Fig. 7C, D) of CO-treated females compared to CO-treated males, though these differences were not statistically significant. E2-treated 5xFAD females did exhibit significantly more CD68 + microglia in the medial cortex (Fig. 6D) relative to CO treated males, though not when compared to CO-treated females, indicating this difference is likely attributable to sex and the 5xFAD genotype rather than E2 treatment. Additionally, CD68 + microglial area was greater (though not significantly so, p = 0.0572) in the 5xFAD E2-treated female subiculum (Fig. 6D) relative to their CO treated male counterparts, though this trend was lost throughout the remainder of the 5xFAD hippocampus, which exhibited no significant differences between groups (Fig. 7C, D). These findings could be an early indication that female microglia are more phagocytic in the 5xFAD, and E2 treatment is not sufficient to masculinize microglial response early in disease. While we observed little to no sex differences or E2-effects on microglial structure and phenotype, E2 treatment could still cause alterations at the molecular level. Therefore, we next sought to investigate potential E2-driven modulation of gene expression.

### E2 treatment subtly shifts gene expression in WT and 5xFAD mice

Despite microglial morphology not directly correlating with a specific activity state, it does indicate to some degree a general microglial response [49]. While E2 masculinization has previously been reported to decrease the number of phagocytic microglia in females to male levels, it is unknown whether these E2 mediated phenotypic shifts are accompanied by changes in gene expression [13, 50]. Given our results thus far, we sought to determine if there were changes in microglial gene expression using a panel of genes known to be differentially regulated between the sexes (Table 1) [16, 51, 52]. We also included genes known to be markers of microglial mediated inflammation, as there are sex-specific increases in inflammation-linked gene transcripts [14]. Although previous studies have examined these genes and their differential regulation, the impact of E2 masculinization is unknown.

**Table 1.** qPCR genes of interest

Category	Gene name	Post-transcriptional function	Reference(s)
Sex-specific	*Akt1s1*	Negative regulator of mTORC1 pathway	He et al., 2016 [56] Villa et al., 2018* [16]
	*Cxcl1*	Chemoattractant for neutrophils	Jiang et al., 2023 [57] Cyr & Vaccari, 2023* [52]
	*IL-1α*	Dual-function cytokine associated with inflammation in disease	Dinarello, 2018 [58] Cyr & Vaccari, 2023* [52] McNaughton & Williamson, 2023 [59]
	*Ccl22*	Dendritic cell/macrophage derived chemokine that binds and activates chemokine receptor CCR4	Vulcano et al., 2001 [60] Cyr & Vaccari, 2023* [52]
	*Cxcl2*	Chemoattractant for neutrophils	Pelus & Fukuda, 2006 [61] Villa et al., 2018* [16]
Inflammatory	*IL-1β*	Dual-function cytokine encoded in the same gene as IL-1α, cleaved into mature form by caspase 1	Dinarello, 2018 [58] Manji et al., 2019* [51]
	*CD36*	Participates in the phagocytosis of apoptotic cells and select pathogens, plays a role in inflammatory responses	Silverstein & Febbraio, 2009 [62] Edler et al., 2021* [63]
	*Cxcl10*	Chemoattractant for activated T cells	Dufour et al., 2002 [64] Manji et al., 2019* [51]
	*Ifnγ*	Cytokine primarily produced by lymphocytes, plays a role in immune response	Kann, Almouhanna & Chausse, 2022 [55]
	*Cxcl12*	Regulator of peripheral hematopoietic stem cells, and key maintenance factor for embryonic and adult neural stem cells	Li et al., 2012 [65]
	*Gfap*	Intermediate filament responsible for forming cytoskeletal structure in glial cells, upregulated in many neurodegenerative diseases	Yang & Wang, 2015 [66] [51]*
	*Trem2*	Transmembrane receptor found on myeloid cells and microglia that plays a complex role in neurodegenerative disease	Ulland & Colonna, 2018 [3]
	*Iba1*	Cytoskeletal protein found in macrophages and microglia, upregulated during microglial activation	Gheorghe et al., 2020 [67] Manji et al., 2019*[51]
X chromosome inactivation	*Eif2s3x*	Encodes translation initiation and elongation factors, known to escape XCI in mice	Filippova et al., 2005 [68]
	*Huwe1*	Functions as an E3 ubiquitin ligase, known to be subject to XCI in mice	Giles & Gill, 2020 [69] Yang et al., 2010 [70]
	*Iqsec2*	GTP binding protein with a domain adjacent to Kdm5c in mouse genome, known to be subject to X inactivation	Yang et al., 2010 [70]
	*Kdm5c*	Lysine demethylase with a domain adjacent to Iqsec2 in mouse genome, known to escape X inactivation	Yang et al., 2010 [70]
	*Kdm6a*	Histone 3 demethylase known to escape X inactivation in mice	Yang et al., 2010 [70] Lin et al., 2025 [71]
	*Xist*	Long non-coding RNA that is essential for X chromosome inactivation in XX eutherian mammals	Cerase et al., 2015 [72] Villa et al., 2018*[16]
	*Ubqln2*	Encodes the ubiquitin-like Ubiquilin, which links ubiquitin to the proteasome; known to be subject to X inactivation in mice	Yang et al., 2010 [70]
H0/DAM	*Clec7a*	Induces microglial activation via Src and Syk family kinases	Xu, Au & Ma, 2022 [73] Brown, 2006 [74]
	*Csf1r*	Encodes a tyrosine kinase growth factor for Csf1, microglial homeostatic marker	Krasemann et al., 2017 [75]
	*Cst7*	Inhibits cysteine proteases, known marker of microglial priming and activation	Baleviciute & Keane, 2023 [76]
	*Itgax*	Leukocyte surface antigen also known as CD11c, known marker of microglial activation	Silvin, Qian & Ginhoux, 2023 [77]
	*P2ry12*	G-protein coupled purinergic receptor, involved in the maintenance of microglial homeostasis	Pitts & Margeta, 2023 [78]

The function of each targeted gene for qPCR, along with source publications. Publications marked with an asterisk report sex-linked differential regulation of the indicated target gene.

**Fig. 8:**
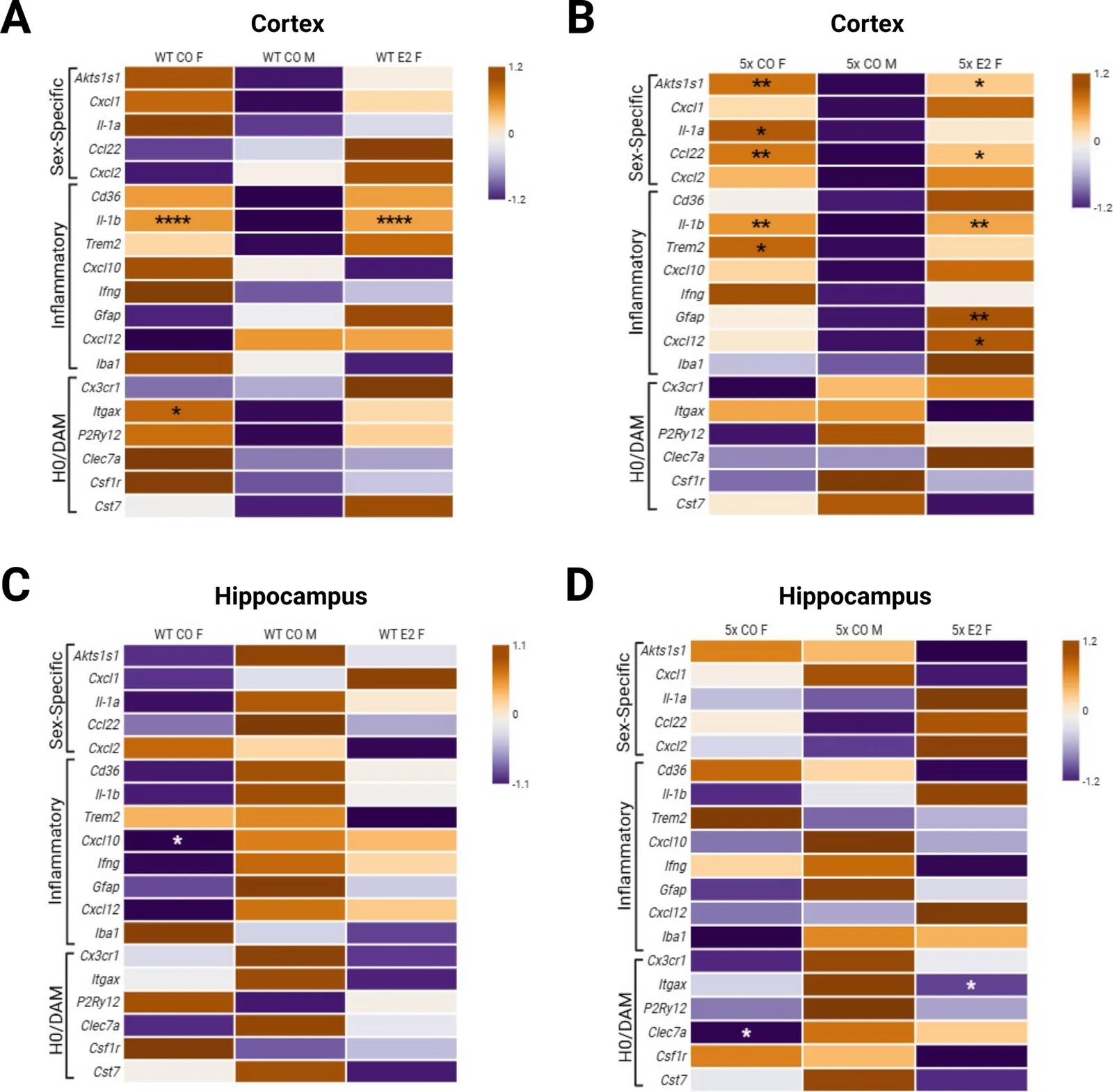
E2 administration alters microglial gene expression in a region-specific and 5xFAD-dependent manner. Heatmaps of cortical gene expression in WT (**A**) and 5xFAD (**B**) mice, and hippocampal gene expression in WT (**C**) and 5xFAD (**D**) mice at 2 months of age. Data are presented as raw ΔCT values normalized across rows. One-way ANOVA of ΔCT values with Tukey’s multiple comparisons post hoc indicated. * p < 0.05, ** p < 0.01, **** p < 0.0001 compared to CO-treated males of each respective genotype. n = 2–15 mice/gene/group. Created in BioRender. Norrell, P. (2026) https://BioRender.com/kg36oz5

Differential gene expression was observed in the cortex of WT and 5xFAD animals, with WT E2 treated females trending towards masculinized patterns of expression, though not significantly for most genes (Fig. 8A, B). *IL-1β* was significantly higher in the cortices of CO- and E2-treated females (F = 68.44, p < 0.0001) compared with CO-treated males, consistent with previously described neuroimmune sex differences [53]. *Itgax* (F = 4.322, p = 0.0244) was also significantly higher in the cortex of CO-treated females compared to CO-treated males, likely attributable to sex differences in immune signaling [54]. Non-significant trends toward masculinization were also present in *Akt1s1*, *IL-1α*, and *Cxcl2* (Supplementary Fig. 6). In 5xFAD cortices, significant differences were present in the expression of *Trem2* (F = 3.520, p = 0.0450)*, Cxcl12* (F = 3.643, p = 0.0409)*, IL-1β* (F = 7.915, p = 0.0027)*, Gfap* (F = 5.538, p = 0.0099), *Akt1s1* (F = 7.243, p = 0.0033)*,* and *Ccl22* (F = 7.632, p = 0.0029)*,* though these differences were not attributable to E2 treatment (Fig. 8B). Additionally, a non-significant trend toward masculinization was present in *Cx3cr1* (Supplementary Fig. 9).

In the WT hippocampus, trends toward masculinization were observed in the expression of *Cxcl1, Iba1, Cxcl12*, and *Cxcl10*, replicating sex-linked differential expression observed in previous studies (Fig. 8C; Supplementary Figs. 10,12) [16, 51, 52, 55]. *Cxcl10* was of particular interest, as relative expression was significantly lower in WT CO-treated females compared to both CO-treated males and E2-treated females (F = 4.563, p = 0.0258), indicative of a potential region-specific shift in the inflammatory microenvironment. Previous studies have indicated that the expression of *Cxcl10* is sexually dimorphic and is upregulated in response to inflammation in female brains [51]. The observed trend toward masculinization with E2 could be the result of microglial re-programming, as the transcriptome shifts towards the male phenotype (Supplementary Fig. 10).

In 5xFAD hippocampi, significant differences were observed in *Clec7a* (F = 3.621, p = 0.0416) and *Itgax* (F = 3.70, p = 0.0375), though, as in the 5xFAD cortex, these differences were not attributable to E2 treatment (Fig. 8D). *Clec7a* did exhibit a non-significant trend toward masculinization alongside *Iba1*, and *CD36* (Supplementary Figs. 11,13). These results suggest E2 induces a subtle (largely non-significant) masculinization of gene expression, particularly in inflammation-linked transcripts, indicative of potential changes to microglial programming consistent with prior characterization of E2 masculinization [14, 15, 43].

Taken together, these findings indicate a subtle, largely non-statistically significant shift in microglial gene transcription, with E2 administration driving trends toward masculinization across all genotypes and brain regions. These findings led us to investigate potential mechanisms responsible for these subtle shifts.

### X chromosome inactivation may underlie E2 masculinization

**Fig. 9:**
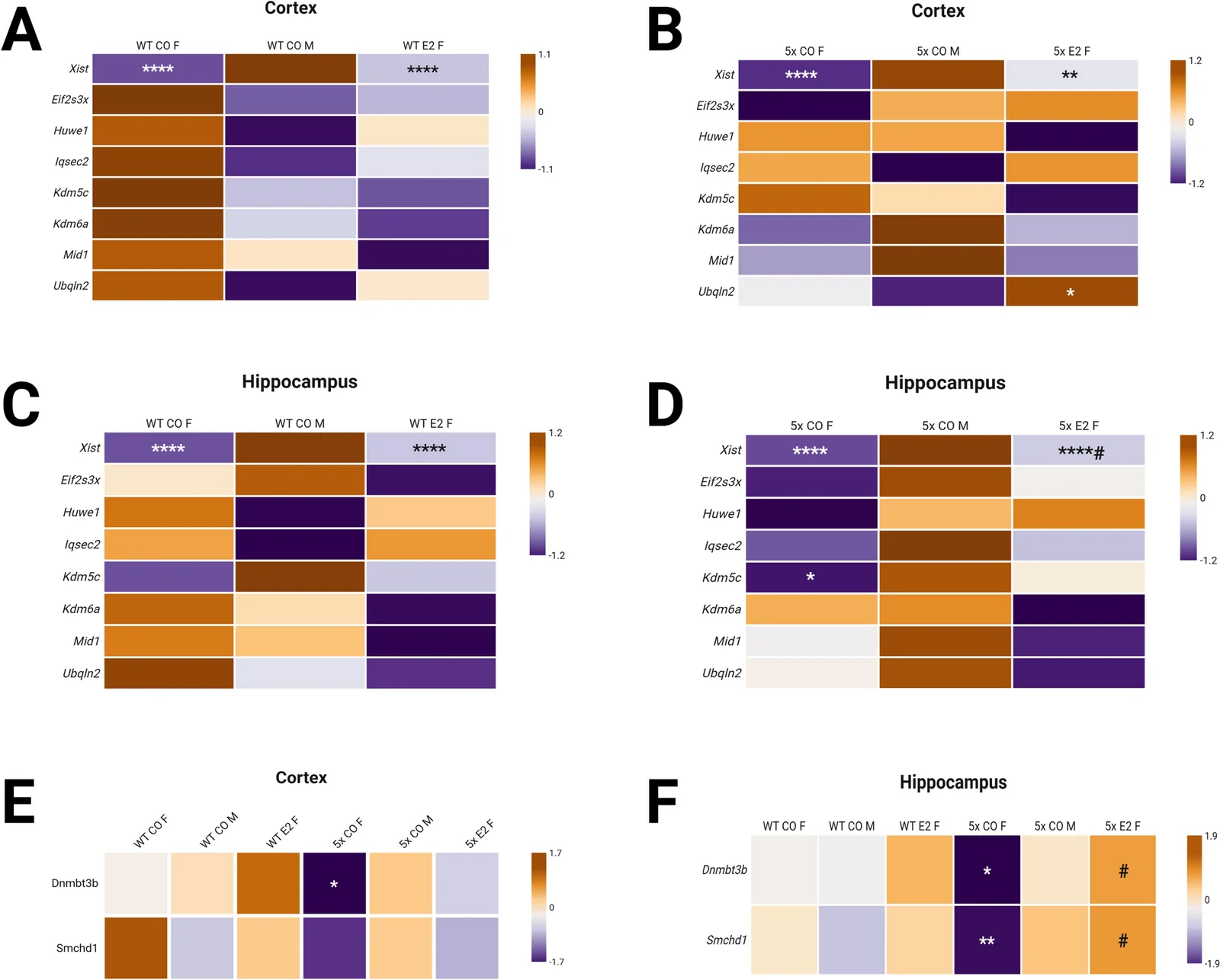
E2 alters X chromosome inactivation profiles. Heatmaps of cortical gene expression in WT (**A**) and 5xFAD (**B**) mice, and hippocampal gene expression in WT (**C**) and 5xFAD (**D**) mice at 2 months of age. Data are presented as raw ΔCT values, normalized across rows. One-way ANOVA of ΔCT values with Tukey’s multiple comparisons post hoc indicated. n = 2–15 mice/gene/group. * p < 0.05, ** p < 0.01, **** p < 0.0001 compared to CO-treated males of each respective genotype. # p < 0.05 compared to 5 × CO females. Heatmap of cortical (**E**) and hippocampal (**F**) chemiluminescent peak area normalized to total protein, normalized across rows. One-way ANOVA of peak area with Tukey’s multiple comparisons post hoc indicated. n = 6–15 mice/region/group. * p < 0.05 compared to 5x CO-treated males. # p < 0.05 compared to 5x CO-treated females. Created in BioRender. Norrell, P. (2026) https://BioRender.com/aovy99p

XCI is an epigenetic regulatory process that occurs in females bearing two X chromosomes to maintain gene dosing. However, inactivation of the X chromosome is never complete, as some genes escape inactivation and are expressed, with many escape genes, such as *Kdm6a* and *Xist*, serving as key regulators of XCI maintenance [71]. Significantly, the X chromosome harbors a high concentration of immune gene transcripts relative to autosomes, with the duplicate copies typically being subject to XCI in females. However, the presence of a second X chromosome has been linked to microgliosis and increased microglial numbers in 5xFAD mice, suggesting escape of immune transcripts involved in microglial activation [19]. This led us to hypothesize that the subtle shifts observed in gene expression could be the result of changes to the molecular machinery involved in XCI. To investigate this hypothesis, we expanded our qPCR analyses to include genes either subject to or known to escape XCI (Table 1), and further investigated two key proteins involved in XCI, Dnmt3b, and Smchd1 (Fig. 9).

*Xist* is a transcriptional marker of X chromosome inactivation that works in conjunction with *Kdm6a* to recruit Dnmt3b and Smchd1 to maintain epigenetic control over XCI [79, 80]. While WT CO-treated females expressed significantly more *Xist* expression than CO-treated males (F = 44.38, p < 0.0001), *Xist* expression was not significantly masculinized in either the cortex or hippocampus (Fig. 9A, C), however, a non-significant trend toward masculinization was present in the hippocampus, along with *Kdm6a*, an upstream regulator of *Xist* (Fig. 9C) [68]. In 5xFAD mice, similar trends were observed in the cortex (Fig. 9B), with CO- and E2- treated females expressing significantly more *Xist* compared to CO-treated males, indicative of a sex difference rather than a treatment effect (F = 19.94, p < 0.0001). *Ubqln2*, a Ubiquilin encoding gene that links ubiquitin to the proteosome, was significantly less in E2 females compared to CO males but not compared to CO females (F = 3.734, p = 0.0375), indicative of a sex difference (Fig. 9B). In 5xFAD hippocampi, however, *Xist* (F = 58.61, p < 0.0001) expression was masculinized, suggesting a region-specific effect of E2 masculinization in 5xFAD mice (Fig. 9D). Differences in hippocampal gene expression were further detected in *Kdm5*c, a known XCI escape gene, with CO-treated females exhibiting significantly more relative to CO treated females. While non-significant, *Kdm5c* also trended toward masculinization in E2 treated females (Fig. 9D) [68]. The region- and genotype-specific upregulation of *Xist* suggests of a larger transcriptomic shift away from the traditional female pattern of X chromosome suppression, and towards a masculinized expression pattern. We next sought to investigate further components of XCI machinery, and investigated two proteins recruited by *Xist*, Dnmt3b and Smchd1.

Dnmt3b is a de novo DNA methyltransferase critical to XCI, functioning as the final step in chromatin conformation to stabilize the chromosome, and is heavily implicated in maintaining the inactive X chromosome (Xi) in females [81, 82]. Smchd1 works in concert with Dnmt3b and is recruited by *Xist* to stabilize and merge heterochromatin compartments (functioning as a structural maintenance of chromosomes (SMC) family protein), thereby modulating XCI [83]. No significant differences in the expression of either protein were present in WT animals (Fig. 9E, F). However, significant differences were present in 5xFAD animals, with Dnmt3b expression being significantly higher in 5xFAD CO female cortices relative to CO-treated males (F = 3.260, p = 0.585) with an accompanying non-significant trend toward masculinization in E2-treated females. In 5xFAD hippocampi, Dnmt3b (F = 5.587, p = 0.0093) and Smchd1 (F = 6.920, p = 0.0042) expression were both significantly masculinized by E2 treatment (Fig. 9F). Together, these findings indicate a shift towards the masculine phenotype with E2 treatment, exhibiting both region and genotype-specific effects.

## Discussion

The current study provides key insights into the molecular underpinnings of the E2 masculinization model and begins to characterize the role of early-life microglial programming beyond the canonical sexually dimorphic nuclei, while also beginning to explore the effects of brain masculinization on disease state outcomes in 5xFAD mice.

We first evaluated changes to the estrus cycle and circulating hormone levels, observing no significant differences attributable to the E2 administration paradigm. The observed skewing of estrus cycle phase distribution is likely attributable to lack of estrus phase monitoring prior to sacrifice and further explains the significantly decreased levels of progesterone observed in 5xFAD E2-treated females, as low-progesterone estrus phases were over-represented in our sample. However, the lack of perturbations to the estrus cycle or circulating hormone levels indicate that the effects of the E2 treatment paradigm likely occur in an ovary-independent manner. This finding is consistent with previous studies of neonatal E2 administration, where subsequent infertility of E2 treated females occurs upstream of the reproductive tract as mPOA synaptogenesis is altered, further driving downstream perturbations in pituitary signaling [84].

We next sought to investigate potential cytoarchitectural changes and found no significant differences across 5xFAD or WT groups, regardless of treatment condition, in the number or percent area coverage of Iba1+ microglia. Further analysis of microglial morphology via Sholl revealed no significant differences in WT animals in either the cortex or hippocampus; however masculinization was observed in the 5xFAD cortex, suggesting a region-specific effect of E2 treatment in 5xFAD animals, as no significant differences in morphology were observed in the 5xFAD hippocampus. Further, we observed a sex-linked increase in CD68+ microglial percent area coverage in 5xFAD CO- and E2- treated females, though significance was only achieved in the E2 treatment group. This finding is potentially notable as the absence of masculinization in CD68+ microglia in the 5xFAD hippocampus suggests E2 treatment may not be sufficient to rescue 5xFAD amyloidosis in early disease, warranting further study of the longitudinal effects of E2 treatment.

As microglial form does not beget function, we next assessed changes in gene transcription. In WT animals, we observed significant differences in cortical expression of *IL-1β* and *Itgax*, and hippocampal expression of *Cxcl10*. The differences in *IL-1β* and *Itgax* were not attributable to E2, but did replicate previous findings regarding sex-linked differential expression [53, 54]. WT hippocampal expression of *Cxcl10*, however, exhibited a trend toward masculinization, which is particularly interesting as *Cxcl10* has been reported to be up-regulated in female brains in response to inflammation, indicating a potential region-specific trend toward masculinization in WT animals, providing further support for immune involvement in brain masculinization [14, 15, 51]. In 5xFAD animals, significant differences were present in the cortical expression of *Cxcl12, Gfap*, *IL-1β*, *Akt1s1*, *Ccl22*, and *Trem2*. These differences werenot attributable to E2 treatment, and are likely attributable to 5xFAD disease pathogenesis, as no significant differences or trends toward masculinization were present [85–91]. In the 5xFAD hippocampus, *Clec7a* was significantly lower in CO-treated females relative to CO-treated males, which is likely attributable to the age of the animals, as *Clec7a* is up-regulated in aged females relative to aged males [92]. *Itgax* was also significantly lower in 5xFAD E2-treated females relative to CO-treated males; however, E2-treated and CO-treated females were not significantly different from each other, indicating this difference is sex-linked and not attributable to E2 treatment. Interestingly, 5xFAD E2-treated females exhibited non-significant trends toward masculinization in the expression of *Cd36*, *Clec7a*, and *Trem2*. These subtle shifts become notable when paired with the CA3 pyramidal cell layer thickness data, where a subtle trend toward masculinization is present, indicating a potential phenotypic shift toward masculinization. Future experiments from our laboratory will continue to investigate these differences in later stages of disease to better understand longitudinal effects of E2 treatment across the lifespan. When considered with the above immunohistochemistry data, these results suggest that E2 masculinization drives differences primarily at the molecular, rather than the structural level.

We next sought to investigate the potential role of E2 masculinization in the modulation of XCI, as the X chromosome shares intimate links to the immune system and microglial activation in 5xFAD mice [19]. We first expanded our qPCR panel to include genes known to escape and be subject to XCI in murines (Table 1). While no significant masculinization occurred in WT animals, trends toward masculinization were present in the cortical expression of two known XCI escape genes, *Kdm5c* and *Kdm6a*. The trend in *Kdm6a* is particularly notable, as Kdm6a is an upstream regulator of *Xist*, suggesting a subtle and non-significant shift in epigenetic regulation of XCI in WT cortices. In WT hippocampi, however, while the trend toward masculinization of *Kdm5c* was lost, it remained present in *Kdm6a*, suggesting a generalized and non-significant effect of E2 masculinization across the cortex and hippocampus of WT animals. In 5xFAD animals, significant masculinization of *Xist* expression was observed, exhibiting regional specificity, as this masculinization was limited to the hippocampus. Interestingly, the masculinization of *Xist* was not accompanied by masculinization (or a trend toward masculinization) of *Kdm6a*, and the mechanism underlying the loss of this trend remains unclear. The trend toward masculinization in *Kdm5c*, however, remained.

These findings led us to further investigate two key proteins recruited by *Xist* and Kdm6a to stabilize the chromosome and maintain proper XCI: Dnmt3b and Smchd1. We observed no significant differences in the expression of either protein in the cortex or hippocampi of WT animals. However, in 5xFAD animals, both proteins were significantly masculinized in the hippocampus, accompanied by a non-significant trend toward masculinization of Dnmt3b in the cortex. While the precise mechanism driving these differences remains unclear, future experiments from our laboratory will continue to evaluate genotype specific effects of masculinization and modulation of XCI to better understand the mechanism underlying these observations. Currently, we hypothesize that the observed masculinization of Dnmt3b and Smchd1 in 5xFAD hippocampi is attributable to epigenetic modifications, as *Xist*, Kdm6a, Dnmt3b, and Smchd1 modulate chromatin accessibility, and thus, gene transcription, in response to chromosomal alignment and circulating hormones. The increased presence of microglia in the 5xFAD hippocampus may also contribute to increasing impacts of masculinization in this region.

These findings are particularly interesting when viewed in conjunction with the observed masculinization of microglial morphology discussed above, as E2 treatment exhibits a region-specific effect in the context of the 5xFAD, with morphological changes occurring in the cortex, while morphology is spared in the hippocampus, with masculinization occurring at the gene expression and protein level, though the underlying mechanism driving this region specificity remains unclear.

Our findings begin to characterize the effects of early life microglial programming and provide a framework for future experiments investigating the impacts of this programming across the lifespan, and in the context of 5xFAD disease pathogenesis. These results also provide critical insight into the effects of E2 masculinization beyond the canonical sexually dimorphic nuclei and reveal a potential novel mechanism of action for the model, as our findings suggest that E2 masculinization drives differences at the molecular and epigenetic level, occurring in a region-specific manner in 5xFAD animals. These results expand on prior knowledge of brain masculinization and lays the groundwork for future experiments investigating the role of XCI in 5xFAD hippocampal neurobiology, and the role of sex hormones therein.

## Supplementary Information

Additional file 1.

## Data Availability

The datasets used and/or analysed during the current study are available from the corresponding author on reasonable request.
