# Supplementary material for: Neonatal estradiol influences regional gene expression for early sex-specific inflammatory responses in the 5xFAD mouse model
Source: Biol Sex Differ. 2026 Jul 3;17:157. doi: 10.1186/s13293-026-00948-z (PMC13621646; doi:10.1186/s13293-026-00948-z)
Supplement: Supplementary file 1 [file 13293_2026_948_MOESM1_ESM.docx]

**Neonatal estradiol influences regional gene expression for early sex-specific inflammatory responses in the 5xFAD mouse model**

**Supplementary Document 1**

**Supplementary Figure 1.** Analysis of subicular NeuN+ percent (left) and cell counts (right) for wild type (top) and 5xFAD (bottom) animals. One-way ANOVA with Tukey’s multiple comparisons *post hoc* revealed no significant differences between groups, indicating neuronal atrophy and cell death had not yet taken place in 5xFAD animals.

**Supplementary Figure 2.** Graphs of Sholl analysis summary metrics in WT cortices, including max intersections (the greatest number of intersections per radii), max intersections radii (the number of radii containing the greatest number of intersections), sum intersections (the total number of intersections across all radii), mean intersections (sum of total intersections divided by the number of radii), branching index (measure of process complexity, measured in single micron steps), intersecting radii (the number of radii containing intersections that cross into one or more additional radii), critical value (maximum value of polynomial fit) and critical radius (the distance from defined center of soma at which the greatest number of intersections occurs). One-way ANOVA with Tukey’s multiple comparisons *post hoc* significance indicated. * p<0.05 compared to WT CO males.

**Supplementary Figure 3.** Graphs of Sholl analysis summary metrics in 5xFAD cortices, including max intersections (the greatest number of intersections per radii), max intersections radii (the number of radii containing the greatest number of intersections), sum intersections (the total number of intersections across all radii), mean intersections (sum of total intersections divided by the number of radii), branching index (measure of process complexity, measured in single micron steps), intersecting radii (the number of radii containing intersections that cross into one or more additional radii), critical value (maximum value of polynomial fit) and critical radius (the distance from defined center of soma at which the greatest number of intersections occurs). One-way ANOVA with Tukey’s multiple comparisons *post hoc* significance indicated. * p<0.05, ** p<0.01, *** p<0.005 compared to 5x CO males and/or 5x CO females.

**Supplementary Figure 4.** Graphs of Sholl analysis summary metrics in CA3 of the WT hippocampus, including max intersections (the greatest number of intersections per radii), max intersections radii (the number of radii containing the greatest number of intersections), sum intersections (the total number of intersections across all radii), mean intersections (sum of total intersections divided by the number of radii), branching index (measure of process complexity, measured in single micron steps), intersecting radii (the number of radii containing intersections that cross into one or more additional radii), critical value (maximum value of polynomial fit) and critical radius (the distance from defined center of soma at which the greatest number of intersections occurs).

**Supplementary Figure 5.** Graphs of Sholl analysis summary metrics in CA3 of the 5xFAD hippocampus, including max intersections (the greatest number of intersections per radii), max intersections radii (the number of radii containing the greatest number of intersections), sum intersections (the total number of intersections across all radii), mean intersections (sum of total intersections divided by the number of radii), branching index (measure of process complexity, measured in single micron steps), intersecting radii (the number of radii containing intersections that cross into one or more additional radii), critical value (maximum value of polynomial fit) and critical radius (the distance from defined center of soma at which the greatest number of intersections occurs). One-way ANOVA with Tukey’s multiple comparisons *post hoc* significance indicated. * p<0.05, ** p<0.01, compared to 5x CO males and/or 5x CO females.

**Supplementary Figure 6.** Individual graphs of ΔCT values of generalized inflammation genes in WT cortices. One-way ANOVA with Tukey’s multiple comparisons *post hoc* significance indicated. **** p<0.0001 compared to WT CO males.

**Supplementary Figure 7.** Individual graphs of ΔCT values of generalized inflammation genes in 5x cortices. One-way ANOVA with Tukey’s multiple comparisons *post hoc* significance indicated. * p<0.05, ** p<0.01 compared to 5x CO males.

**Supplementary Figure 8.** Individual graphs of ΔCT values of DAM and homeostatic genes in WT cortices. One-way ANOVA with Tukey’s multiple comparisons *post hoc* significance indicated. * p<0.05 compared to WT CO males.

**Supplementary Figure 9.** Individual graphs of ΔCT values of DAM and homeostatic genes in 5x cortices. One-way ANOVA with Tukey’s multiple comparisons *post hoc* significance indicated. * p<0.05 compared to 5x CO males.

**Supplementary Figure 10.** Individual graphs of ΔCT values of generalized inflammation genes in WT hippocampi. One-way ANOVA with Tukey’s multiple comparisons *post hoc* significance indicated. * p<0.05 compared to WT CO males.

**Supplementary Figure 11.** Individual graphs of ΔCT values of generalized inflammation genes in 5x hippocampi. One-way ANOVA with Tukey’s multiple comparisons *post hoc* revealed no significant differences between groups.

**Supplementary Figure 12.** Individual graphs of ΔCT values of DAM and homeostatic genes in WT hippocampi. One-way ANOVA with Tukey’s multiple comparisons *post hoc* revealed no significant differences between groups.

**Supplementary Figure 13.** Individual graphs of ΔCT values of DAM and homeostatic genes in 5x hippocampi. One-way ANOVA with Tukey’s multiple comparisons *post hoc* significance indicated. * p<0.05 compared to 5x CO males.

**Supplementary Figure 14.** Individual graphs of ΔCT values of canonical XCI escape and inactivation genes in WT cortices. One-way ANOVA with Tukey’s multiple comparisons *post hoc* significance indicated. **** p<0.0001 compared to WT CO males.

**Supplementary Figure 15.** Individual graphs of ΔCT values of canonical XCI escape and inactivation genes in 5x cortices. One-way ANOVA with Tukey’s multiple comparisons *post hoc* significance indicated. * p<0.05, ** p<0.01, **** p<0.0001 compared to 5x CO males.

**Supplementary Figure 16.** Individual graphs of ΔCT values of canonical XCI escape and inactivation genes in WT hippocampi. One-way ANOVA with Tukey’s multiple comparisons *post hoc* significance indicated. **** p<0.0001 compared to WT CO males.

**Supplementary Figure 17.** Individual graphs of ΔCT values of canonical XCI escape and inactivation genes in 5xFAD hippocampi. One-way ANOVA with Tukey’s multiple comparisons *post hoc* significance indicated. * p<0.05, ** p<0.01, **** p<0.0001 compared to 5xFAD CO males and/or 5x CO Females.

**Supplementary Figure 18.** Individual graphs of chemiluminescent peak area normalized to total protein in WT and 5xFAD cortices and hippocampi. One-way ANOVA with Tukey’s multiple comparisons post hoc significance indicated. WT and 5xFAD groups analyzed separately to maintain statistical power. * p<0.05, ** p<0.01 compared to 5xFAD CO treated males and/or 5xFAD CO treated females.
